# The origin of the cooperativity in the streptavidin-biotin system: A computational investigation through molecular dynamics simulations

**DOI:** 10.1038/srep27190

**Published:** 2016-06-01

**Authors:** Fengjiao Liu, John Z. H. Zhang, Ye Mei

**Affiliations:** 1 State Key Laboratory of Precision Spectroscopy, School of Physics and Materials Science, East China Normal University, Shanghai 200062, China; 2 Department of Physics, School of Physics and Materials Science, East China Normal University, Shanghai 200062, China; 3 NYU-ECNU Center for Computational Chemistry at NYU Shanghai, Shanghai 200062, China

## Abstract

Previous experimental study measuring the binding affinities of biotin to the wild type streptavidin (WT) and three mutants (S45A, D128A and S45A/D128A double mutant) has shown that the loss of binding affinity from the double mutation is larger than the direct sum of those from two single mutations. The origin of this cooperativity has been investigated in this work through molecular dynamics simulations and the end-state free energy method using the polarized protein-specific charge. The results show that this cooperativity comes from both the enthalpy and entropy contributions. The former contribution mainly comes from the alternations of solvation free energy. Decomposition analysis shows that the mutated residues nearly have no contributions to the cooperativity. Instead, N49 and S88, which are located at the entry of the binding pocket and interact with the carboxyl group of biotin, make the dominant contribution among all the residues in the first binding shell around biotin.

Cooperativity of residues in modulating the affinity of protein-ligand binding has been frequently used by experimental chemists^1^, despite that this concept lacks of a solid theoretical definition^2^,^3^,^4^. Through double-mutant cycles, the excess loss of binding affinity in double mutant relative to the sum of that for two single mutants has been considered as a direct evidence of the cooperativity between these two mutated residues^5^. It is believed that neutral residues within 7 Å or charged residues within 10 Å in the receptor may have significant cooperativity in modulating its binding affinity to ligands^5^,^6^.

Streptavidin-biotin complex is one of the strongest binding partners occurring in nature and is far beyond the normal protein-ligand binding strength^7^,^8^,^9^ and has been widely used in biochemical sensing applications, especially in the identification of possible new drug targets^10^. As shown in Fig. 1, biotin is confined in the active site of streptavidin by eight hydrogen bonds, as well as van der Waals interaction among non-polar groups^11^. Among these “first shell” residues, five of them bind to the biotin ureido group and are deeply buried inside the pocket. The side chains of Asn23, Ser27, and Tyr43 form hydrogen bonds with the ureido oxygen atom. Ser45 and Asp128 interact with the ureido H atoms from two sides. Previous molecular dynamics simulations and energy decomposition studies^12^ suggested that the dominant contribution to this strong binding affinity was from van der Waals interaction between the ligand and the non-polar groups, especially the tryptophan residues, encompassing the ligand. Strong electrostatic and hydrogen bond interactions between the ligand and the receptor are largely canceled out by the penalty for removing water molecules from the binding site and the desolvation of the ligand. However, some recent studies have identified the significant contribution from electronic polarization effect, which had not been taken into account explicitly in Kollman’s work^13^,^14^. Besides, some residues that bind to the “first shell” residues but not to biotin may also play an important role in modulating the binding affinity through the hydrogen bond cooperativity^14^,^13^. In addition, a flexible loop (Ser45 to Ser52) connecting *β* strands 3 and 4 (L3/4)^15^,^16^, facilitates ligand binding by excluding solvent molecules from the active site, enhancing the direct hydrogen-bonding interactions and hydrophobic interactions with the loop closed. It could cause 27.1 kcal/mol free energy increase from the loop’s open state to closed state, which is estimated by General and Meriovitch using the hypothetical scanning molecular dynamics (HMSD) method^17^,^18^.

Some studies focusing on the hydrogen bond network show that the streptavidin biotin hydrogen bonds indeed display exceptionally large free energy alterations after mutation^19^,^20^,^21^,^22^. Freitag *et al*. pointed out that D128 acts as an intermediate on the way of biotin dissociation by a series of site-directed mutagenesis, biophysical, and X-ray crystallographic approaches^23^. A number of studies on S45A mutant indicated that the S45A mutation exhibits a 1,700-fold greater dissociation rate and 907-fold lower equilibrium affinity for biotin relative to the wild-type streptavidin at 37 °C, exhibiting its important role in modulating both the equilibrium and transition state energetics^22^. Isothermal titrating calorimetry (ITC)^24^ experiments performed on these mutants by Hyre *et al*. indicated that the double-mutant resulted in 10.1 kcal/mol loss of binding affinity, while that caused by mutation of S45A and D128A single-mutations were just 4.3 and 4.4 kcal/mol, respectively. This result demonstrated that the loss of binding affinity in the S45A/D128A double-mutant (DM) was not just a linear combination of single-mutant energetic perturbations but showed a cooperative effect. They suggested that this effect is caused by the structural rearrangements at the S45 position when the D128 carboxyl group is removed, which masks the true energetic contribution of the D128-biotin interaction^11^. However, the structural differences among the wild-type and the mutants are very small, which can be seen in Fig. 2 and qualitatively from Table 1. These data indicate that the cooperativity does not come from the global structure rearrangement after these mutations.

Although crystallographic and calorimetric experiments have proposed a conjecture that these pivotal hydrogen bonds act cooperatively, direct evidence from computer simulation has not yet been presented. A large number of studies have shown that with proper parameterization molecular mechanics (MM) is quite successful in modeling biological molecules, such as proteins and nucleic acids^25^,^26^,^27^,^28^,^29^,^30^. Nevertheless, in standard force fields, e.g., CHARMM^26^ and AMBER^31^,^32^,^33^,^34^, the atomic charge magnitudes do not change in response to the chemical environment. Therefore, they only give a mean-field representation of the charge distribution, and fail to capture the adaptation of electron density to the environment, which is highly inhomogeneous and protein-specific. Electronic polarization is critical for an accurate description of electrostatic interaction, due that the electrostatic interaction depends not only on their distance of atoms but also on their locations in the protein and their local solvent environment^35^,^36^,^37^,^38^. Cooperativity is a typical nonadditive phenomenon, which poses a challenge to traditional force field. Ji *et al*. proposed the polarized protein-specific charge (PPC) scheme^38^ that can reflect the specificity of charge distribution in a certain protein, based on a quantum mechanical fragmentation method termed the molecular fractionation with conjugate caps. This charge scheme is further refined by Zeng *et al*. via improving its numerical stability^39^. The viability and effectiveness of this charge model have been proved in several studies^40^, including biotin and its analogues binding to the wild type (strept) avidin and the mutant^14^,^41^,^42^,^43^. Meanwhile, quantum mechanical calculation has been applied to this system by Houk *et al*., and they found that these hydrogen bonds act cooperatively^13^. The charged D128 residue provides the driving force for the cooperativity in the hydrogen-bonding network by greatly polarizing the ureido of biotin. If the D128 residue is mutated, the hydrogen bond network connecting the “first shell” and the “second shell” residues is disrupted. Unfortunately, due that QM calculation is too costly for the whole protein, the thermal effect had not been taken into consideration in their study.

When structure ensemble is available, the free energy can be estimated using various methods differ by their rigor and speed. All these methods can be roughly classified into two categories, i.e. the pathway method and the end-state method^44^. As suggested by the name, pathway methods compute the sum of small changes along a geometrical or alchemical route connecting the initial and the final states, while end-state methods consider the conformations of the free and the bound states only and compute the binding affinity by taking a difference. No intermediate states are considered in end-state methods. The thermodynamic perturbation (TP)^45^, thermodynamic integration (TI)^46^,^47^,^48^ and Bennett acceptance ratio (BAR)^49^,^50^ methods are the typical representatives of pathway methods. The end-state methods usually refer to the linear interaction energy (LIE)^51^,^52^ and the molecular mechanics/Poisson-Boltzmann with surface area (MM/PBSA) method^53^,^54^,^55^, along with its variant utilizing the generalized Born solvation model (MM/GBSA). Although the pathway methods can provide results within 1 to 2 kcal/mol from the experimental measurement, their computational expenses are usually considerably higher than that of the end-state methods^44^. The LIE method requires a training process and is more suitable for the calculation of the (relative) binding free energies of a series of ligands^52^. The (strept)avidin-biotin system has been widely studied using several methods, including FEP^56^, LIE^57^, and MM/PBSA^58^,^59^,^60^,^61^. All of the computational results showed good correlation with the experimental values^62^. Therefore, the MM/PBSA method was utilized in this work for the calculation of the binding affinities.

In this work, classical molecular dynamics (MD) simulations have been carried out to study the wild-type streptavidin and its D128A, S45A and S45A/D128A mutants complexed with biotin. The electrostatic polarization effect is taken into account by utilizing the PPC scheme. The binding free energies are estimated using the MM/PBSA method. The results show that this cooperativity comes from both the enthalpy and entropy contributions. And the former contribution mainly comes from the alternations of solvation free energy.

## Methodology

### Molecular dynamics simulation

The initial structures for the wild-type, S45A, D128A and S45A/D128A streptavidin, which will be denoted as WT, S45A, D128A and DM hereafter, were downloaded from Protein Data Bank (entry: 1MK5, 1DF8, 1SWT and 1MEP, respectively). Streptavidin is a dimer-of-dimer, and the complete structures were generated by applying the symmetry operation with Discovery Studio Visualizer except for DM, of which the coordinates for all the four units are available in the crystal structure. The coordinates of the missing residues are predicted by the ModLoop server^63^,^64^. AMBER03 force field^65^ and the general AMBER force field (GAFF)^66^ were applied to the protein and ligand, respectively. All hydrogen atoms in protein were automatically added by the LEaP module in AMBER package. Each complex was soaked in a periodic box of TIP3P water^67^, with the minimal distance between the complex and the boundary of the box set to 12 Å. Sodium ions were added to neutralize the charge. Crystal water molecules were reserved when preparing the system. Each system was first optimized with weak restraint exerted on the ligand molecules and the proteins (excluding those missing residues optimized by the ModLoop server) and then was fully relaxed until the energy gradient was below 1.0 × 10^−4^ kcal/(mol Å). With this energy-minimized structure, the PPCs were fitted using the molecular fractionation with conjugate caps/Poisson–Boltzmann (MFCC/PB) method^38^,^39^. The quantum mechanical calculations were carried out at B3LYP/6-31G(d) level using Gaussian 09^68^. The calculated atomic charges were then averaged over the four chains and were used in the subsequent MD simulations. Parameters other than the atomic charges were kept intact. Each system was gradually heated up to 300 K in 100 ps, and then further relaxed for 20 ns at NPT ensemble. The temperature was regulated with the Langevin dynamics heat coupling scheme and the collision frequency was set to 2.0 ps^−1^. The pressure relaxation time was set to 1.0 ps. SHAKE algorithm was applied to restrain all covalent bonds involving hydrogen atoms^69^. The integral time step was set to 2 fs. The van der Waals (vdW) interaction was truncated at 10 Å. Long range Coulomb interaction was calculated using particle mesh Ewald (PME) with 10 Å cutoff in real space^70^.

### MM/PBSA calculation of the binding free energies

The binding affinity was calculated by^44^


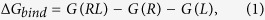


where *R* and *L* stand for the receptor and the ligand. The free energy of each reactant was estimated as


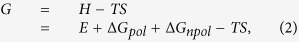


with


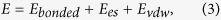


where *E*_*bonded*_, *E*_*es*_ and *E*_*vdw*_ are the bonded energy, the Coulomb interaction energy and the vdW interaction energy, respectively. Δ*G*_*pol*_ is the polar solvation free energy, which was calculated by solving the Poisson-Boltzmann equation. Δ*G*_*npol*_ is the nonpolar contribution to the solvation free energy, which was approximately estimated as a function of the solvent-accessible surface area (SASA) as *G*_*npol*_ = *γA*_*s*_ + *b* with γ = 0.00542 kcal/(mol · Å^2^) and b = 0.92 kcal/mol. The surface area *A*_*s*_ was calculated with the molsurf package^71^. *S* is the entropy contribution estimated using the normal-mode approximation. The binding free energy is finally expressed as


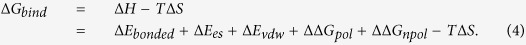


A 20-ns production MD simulation was performed for each system. However, the hydrogen bonding network in chain A of S45A was not as stable as those in other chains after about 17 ns. Therefore, the last 3-ns trajectory was abandoned for the S45A mutant. All of the terms in Eq. 4 were averaged over the snapshots extracted from the most stable MD trajectory, i.e. 4–20 ns for WT, D128A and DM, and 4–17 ns for S45A. Altogether, eight hundred snapshots were used for the calculation of enthalpy. The “mbondi2” radii were used in the PB calculations, and the grid spacing was set to 0.5 Å. The exterior and interior dielectric constants were set to 80 and 1, respectively. One hundred snapshots were used for the entropy calculation using the Nmode program. All the molecular dynamics simulations and MM/PBSA analysis were conducted with Amber12 and AmberTools package.

## Result and Discussion

### Structure stability

**Figure 3:**
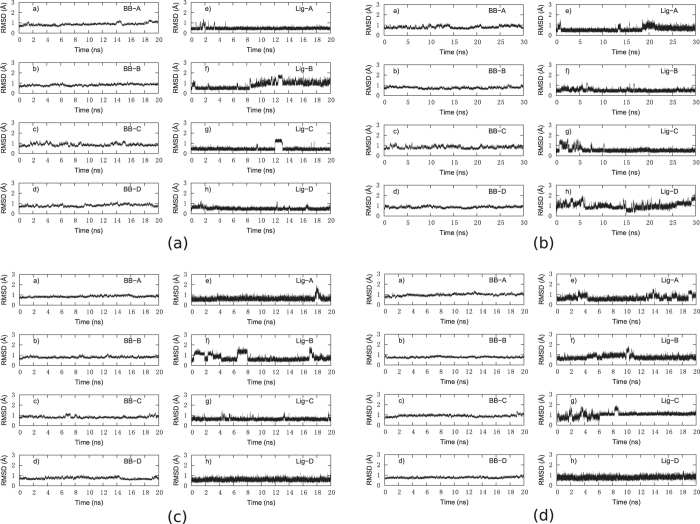
RMSD of the backbone (BB) and the biotin (Lig) atoms along the simulation for (**a**) WT, (**b**) S45A, (**c**) D128A and (**d**) DM. Chains are labeled by A to D.

**Figure 4:**
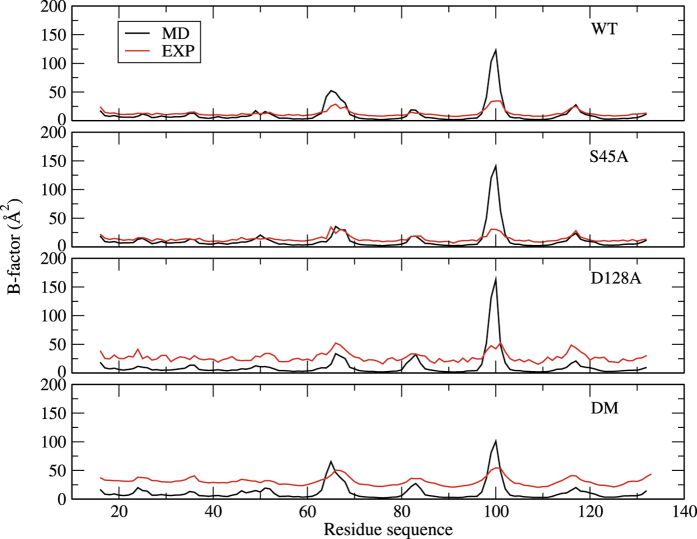
The average B-factor values of the residues.

**Figure 5:**
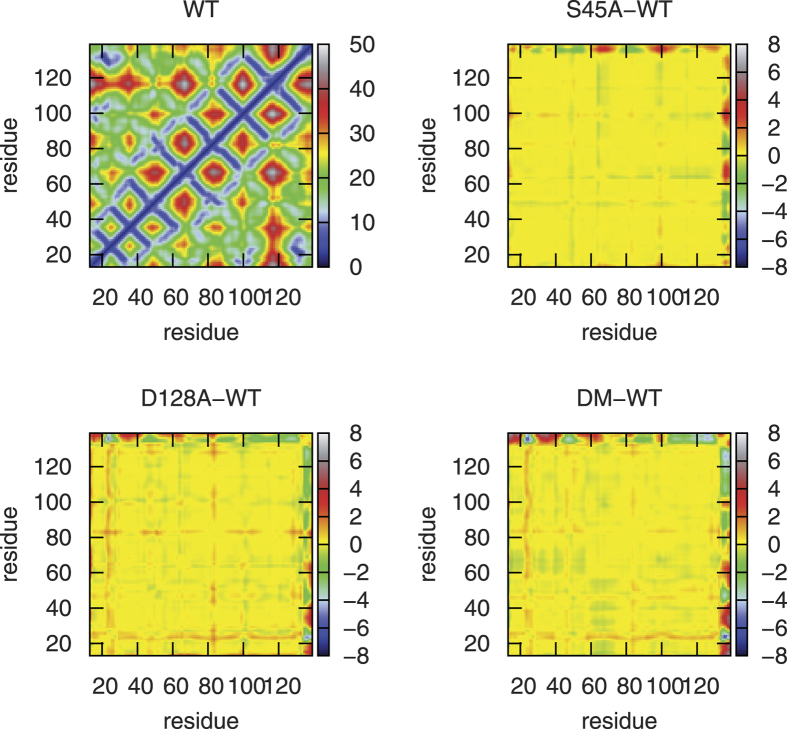
The contact maps which show the interaction between the residues of protein. (top right) The difference map between S45A and WT. (bottom left) The difference map between D128A and WT. (bottom right) The difference map between DM and WT.

The overall structure variation of the protein and ligand molecules during the whole simulation is shown in Fig. 3. The backbone RMSDs of the proteins are always around 1 Å with very small fluctuations, indicating remarkable stabilities of the protein scaffolds. Besides, significant variation of conformation or orientation for ligand in the binding pocket of the four system has not been detected. The RMSDs of biotin in the binding pocket are marginally larger than those of the protein backbone, but are still blow 2 Å in majority of the simulation time. Due to the slightly different conformations of the four chains and the random velocities assigned to the atoms at the beginning of the simulations, notable differences among the four chains can be observed, especially for the ligand RMSDs. The simulated and experimental B-factors are shown in Fig. 4. They only qualitatively agree with each other. This is not unexpected, because the fluctuations of the proteins in crystal are significantly impeded by packing interaction of neighboring units. Nonetheless, the flexible regions can be detected from both the simulated and experimental data. The crystal water molecules are retained when building the systems. Some of them are located in the biotin binding pocket and are close to residue 128. However, these water molecules are not that stable during the simulations of D128A and DM. Frequent exchange with surrounding water molecules can be observed. Furthermore, no significant change in the internal structure of the protein can be detected, which can be read from the contact map shown in Fig. 5 defined as the average distance between *C*_*α*_ atoms. Compared with WT, S45A leads to only small alternations of the distance between nonterminal *C*_*α*_ atoms, which are generally limited within ±1 Å. Due to the large flexibility of the C-terminal residues, they show considerable change of the distances to all the other residues. However, it is believed that these terminal residues have little impact on the binding affinity of the ligand, because their interaction with the ligand are strongly screened by solvent molecules. Relatively large difference can be seen for Q24 and Y83 in D128A and DM. These residues are located in the loop regions of the protein, which show great flexibility as indicated by the B-factors shown in Fig. 4.

**Figure 6:**
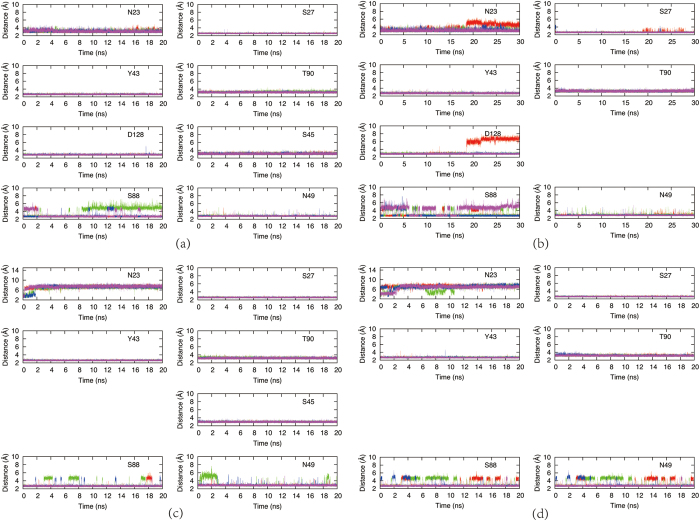
The bond length variations of those critical hydrogen bonds which are directly bound to biotin along the simulations for (**a**) WT, (**b**) S45A, (**c**) D128A and (**d**) DM. N23, S27, Y43, T90, D128, S45, S88, N49 are bound to biotin. Chain A, B, C, D are colored in red, green, blue and purple, respectively. White spaces are left for clarity.

**Figure 7:**
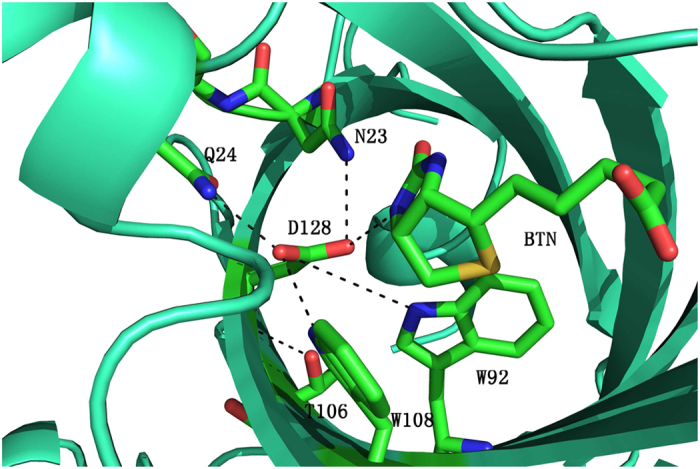
The local environment of residue D128 in the WT system. The hydrogen bonds to D128 are marked as dashed lines.

Interaction of the hydrogen bonds between streptavidin and biotin is essential for the binding affinity, of which the strength can be indirectly inspected by the variation of bond length as shown in Fig. 6. The carboxyl group of biotin is partially exposed to the solvent. Invasion of water molecules destabilizes the hydrogen bonds between this carboxyl group and the proteins (N49 and S88). Therefore, these two hydrogen bonds are interrupted and reconstructed repeatedly during the simulations. In WT, all other hydrogen bonds between the ligand and the protein are extremely stable and are consistent among all the chains. The largest fluctuation is seen for the hydrogen bond connecting the ligand and N23. N23 is anchored near biotin by the hydrogen bond between N23 and D128 (see Fig. 7), as well as the direct hydrogen bonding interaction between N23 and the ligand. When D128 mutates into alanine as in D128A and DM, the side chain of N23 can no longer maintain this orientation and its hydrogen bond to biotin broke quickly at about 2 ns. In S45A, this hydrogen bond in chain A broke after 17 ns, but were very stable in other chains. This inconsistency might result from the limited accuracy of the force field utilized. Snapshots were extracted from the 4–17 ns trajectory of S45A for the free energy calculation, while for the other systems the snapshots are taken from 4–20 ns.

### MM/PBSA analysis of binding affinity

The results from single trajectory MM/PBSA analysis are shown in Table 2. Due to the existence of several hydrogen bonds, WT has a strong Coulomb interaction (−103.64 kcal/mol) with biotin, of which about three fourths (76.33 kcal/mol) is canceled by the polar desolvation free energy. The total electrostatic term of enthalpy is −27.31 kcal/mol, which is about the same magnitude as the vdW interaction (−30.76 kcal/mol). The nonpolar solvation free energy contributes to the binding affinity by −3.37 kcal/mol for WT, which is almost invariant in all the four systems. The entropy loss (*T*Δ*S*) is also very consistent among all the system, which is in the range from 18.63 to 20.27 kcal/mol. Without considering the penalty from loop closing, the binding free energy of biotin to WT is −41.17 kcal/mol with a standard error of 0.36 kcal/mol. This result is consistent with our previous study^14^. From WT to S45A, a hydrogen bond is eliminated, leading to an 8.58 kcal/mol loss of Coulomb interaction between the ligand and the receptor, accompanied by a 2.74 kcal/mol drop of polar dehydration free energy. The net loss of binding enthalpy and total binding free energy are 5.55 and 5.27 kcal/mol, respectively, which are very close to the experimental measurement (6.10 and 4.30 kcal/mol). The strong Coulomb repulsion between the carbonyl groups in biotin and D128 is removed after the D128A mutation, which results in an enhancement of Coulomb interaction by −25.08 (−128.72 vs. −103.64) kcal/mol. Nonetheless, the desolvation penalty increases by 32.02 kcal/mol. The net loss of binding enthalpy and total binding free energy are 8.49 and 6.85 kcal/mol, respectively, which are about 2.5 kcal/mol larger than the experimental values (5.90 and 4.40 kcal/mol). From S45A to DM, the Coulomb interaction is strengthened by 29.33 kcal/mol, which is larger than that from WT to D128A. In concert, the increase of dehydration penalty is 37.51 kcal/mol. The total binding free energy of biotin to DM is −26.37 kcal/mol, which is 14.80 kcal/mol weaker than that to WT. This decrease in binding affinity shows an extra loss of 2.68 kcal/mol relative to the direct sum of two single mutations, indicating a cooperative characteristics of S45 and D128 in binding to biotin.

Within this extra loss of binding free energy, 1.54 kcal/mol comes from the enthalpy and 1.14 kcal/mol comes from the entropic contribution (See Table 3). Although the mutations lead to the annihilations of the hydrogen bonds to the ligand, the gas phase interaction, which is defined as the sum of Δ*E*_*ele*_ and Δ*E*_*vdw*_, cannot explain this cooperativity. Indeed, it gives a wrong sign (−3.92 kcal/mol). The enthalpic contribution to this cooperativity comes from the solvation free energy (5.46 kcal/mol), which is a global property. This result does not agree with the experimental decomposition of the contributions, which shows that the entropy change is the main source of the cooperativity. We believe that the inconsistency originates from the dissimilar definitions of entropy in the experiment and in the MM/PBSA analysis. In the former, the entropy includes the contributions from both of the complex and the solvent molecules. While in MM/PBSA analysis, the solvent contribution to the entropy is merged into the nonpolar solvation term.

### Free energy decomposition

In order to obtain the fingerprints of the interaction between the ligand and the residues, pairwise energy decompositions of enthalpy were carried out for the eight residues which directly interact with biotin in the WT system^72^,^73^. The results are listed in Table 4. The interaction between biotin and the 45^th^ residue decreases in S45A for losing one hydrogen bond, while the interaction is strengthened in D128A as the biotin moves towards residue S45 once it loses the attraction from residue D128. This can be evidenced by the distance variation between S45 and biotin as shown in Table S1 in Supplementary Information and Fig. 6. The average hydrogen bond length in D128A is 3.0 Å, which is slightly shorter than that in WT (3.1 Å). On the one hand, regardless of whether the 128^th^ residue is aspartate or alanine, this S45A mutation leads to a loss of binding affinity between the 45^th^ residue and biotin of about 3.2 kcal/mol. As a result, the cooperativity comes from residue S(A)45 is merely −0.08 kcal/mol. On the other hand, the attraction between D(A)128 and biotin is immune to the mutation of S45A. D128 has a strong (anion–dipole) interaction with biotin, and bridges the first and the second binding shells by forming a hydrogen bond network with several residues around. The structure near D128 is very rigid and the S45A mutation has no significant impact on the interaction pattern between D128 and biotin. In WT and S45A, the contributions to the binding affinity from D128 are −14.00 and −13.90 kcal/mol, respectively. After the D128A mutation, A128 has only marginal contribution to the binding affinity (−0.20 kcal/mol in D128A and −0.15 kcal/mol in DM). The variation of interaction between N23 and biotin is correlated with that between D128 and biotin as the hydrogen bond between N23 and biotin disappears once D128 mutates into alanine. This is in accordance with the distances of hydrogen bonds, which are 3.1, 3.2, 7.4, and 7.1 Å in WT, S45A, D128A, and DM respectively (see Table S1). The cooperativity originating from D(A)128 and N23 are −0.05 kcal/mol and −0.07 kcal/mol, respectively. S27 forms a hydrogen bond with the ureido group in biotin, and its contributions to the binding affinity in all the mutants are similar and are 1.5 to 2.0 kcal/mol smaller than that in WT. It contributes −2.16 kcal/mol to the cooperativity. Y43 shows a similar characteristic and contributes −1.30 kcal/mol to the binding affinity. The contribution of T90 to the binding affinity decreases in the single mutants as compared to that in WT, but increases a little bit in DM. It contributes to the cooperativity by −1.87 kcal/mol. Residues N49 and S88 have the largest contribution to the cooperativity (5.70 kcal/mol and 2.43 kcal/mol respectively). From D128A to DM, the S45A mutation leads to a loss in binding affinity of 1.69 kcal/mol from N49. However, from WT to S45A, the S45A mutation leads to an enhanced interaction between N49 and biotin. This enhancement of attraction is caused by the strengthened interaction between the carboxyl group of biotin and the amide group of N49 as the amide group switches towards the carboxyl group in biotin occasionally and forms a hydrogen bond between them. A direct evidence is the distance between the carbon atom in the carboxyl group of biotin and the nitrogen atom in the amide group of N49 showing in Table S2. The distance becomes shorter obviously in S45A than in the other complexes. The contribution from S88 is quite close in WT, S45A and DM, but is strengthened in D128A. This is in accordance with the hydrogen bond length listed in Table S1. N49 and S88 form hydrogen bonds with the carboxyl group of biotin and are susceptible to the depth of biotin buried in the pocket. All these changes mainly come from the pairwise electrostatic interaction between the ligand and the residues (see Table S3 in Supplementary Information). The biotin molecule itself makes a cooperativity contribution of −2.20 kcal/mol, due to the variation of desolvation penalty.

## Conclusion

Hydrogen bondings in the streptavidin-biotin complex show strong cooperativity. Experiments have shown that S45A/D128A double mutation leads to excess loss of binding affinity than the direct sum of the dual single mutations. In this work, molecular dynamics simulations have been carried out to uncover the origin of the cooperativity. The structures of the wild-type streptavidin-biotin complex and the mutants are stable during the simulations. Only marginal alternations of the internal structural relaxation can be detected after these mutations. Free energy calculations show that the change of the gas phase interaction is not the source of the cooperativity, and it mainly originates from the solvation free energy and the entropic contributions. Interaction fingerprints show that mutations cause the change of the interaction not only between the ligand and the mutated residues, but also that between the ligand and the conserved residues. The residue-decomposed free energy picture shows that the enthalpy contribution to the cooperativity mainly comes from N49 and S88, which interact with the carboxyl group of biotin. It is the strengthened interaction in one of the single mutants between biotin and these two residues that results in a positive contribution to the cooperativity. All these results indicate a synergistical feature of the hydrogen bonding in the streptavidin-biotin complexes.

## Additional Information

**How to cite this article**: Liu, F. *et al*. The origin of the cooperativity in the streptavidin-biotin system: A computational investigation through molecular dynamics simulations. *Sci. Rep.* **6**, 27190; doi: 10.1038/srep27190 (2016).

## Supplementary Material

Supplementary Information

## Figures and Tables

**Figure 1:**
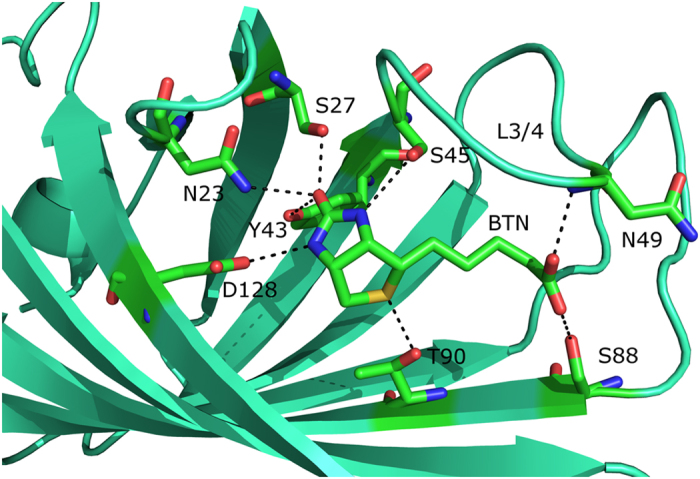
Binding of biotin to the wild-type streptavidin. The key hydrogen bonds are marked as dashed lines.

**Figure 2:**
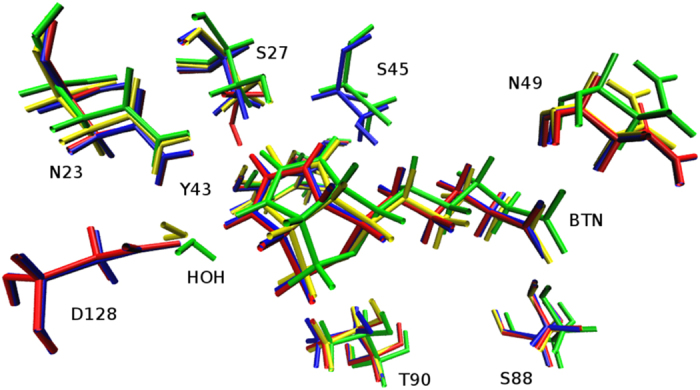
Superposition of the wild-type (blue), S45A (red), D128A (green) and S45A/D128A (yellow) biotin complexes in the biotin binding site.

**Table 1:** The root mean square deviation of the backbone atoms in the mutants from WT.

	RMSD (Å)
S45A	0.13
D128A	0.55
DM	0.28

**Table 2:** Binding free energy components in a unit of kcal/mol. The standard errors of the total binding free energies are also shown.

	Δ*E*_*ele*_	Δ*E*_*vdw*_	Δ*E*_*np*_	Δ*E*_*pb*_	Δ*E*_*es*_	Δ*H*	−*T*Δ*S*	Δ*G*
WT	−103.64	−30.76	−3.37	76.33	−27.31	−61.44	20.27	−41.17 ± 0.36
S45A	−95.06	−31.02	−3.40	73.59	−21.47	−55.89	19.99	−35.90 ± 0.36
D128A	−128.72	−29.13	−3.45	108.35	−20.36	−52.95	18.63	−34.32 ± 0.37
DM	−124.39	−29.06	−3.51	111.10	−13.29	−45.86	19.49	−26.37 ± 0.37

**Table 3:** The calculated and experimental variations of binding free energy (kcal/mol) relative to WT.

System	MM/PBSA								ITC		
	Δ*G*	ΔΔ*G*	Δ*H*	ΔΔ*H*	ΔΔ*H*_*gas*_	ΔΔ*G*_*sol*_	−*T*Δ*S*	−*T*ΔΔ*S*	ΔΔ*G*	ΔΔ*H*	−*T*ΔΔ*S*
WT	−41.17		−61.44				20.27		0.0	0.0	0.0
S45A	−35.90	5.27	−55.89	5.55	8.32	−2.77	19.99	−0.28	4.3	6.1	−1.8
D128A	−34.32	6.85	−52.95	8.50	−23.45	31.94	18.63	−1.64	4.4	5.9	−1.5
DM (predicted)^1^	−29.05	12.12	−47.39	14.05	−15.13	29.17	18.35	−1.92	8.7	12.0	−3.3
DM (calculated)^2^	−26.37	14.80	−45.86	15.59	−19.05	34.63	19.49	−0.78	10.1	10.3	−0.3
Cooperativity^3^		2.68		1.54	−3.92	5.46		1.14	1.4	−1.6	3.0

^1^The loss of binding affinity predicted by a direct sum of single-mutant energetic perturbations (Δ*E*_*WT*_ + ΔΔ*E*_*S*45*A*_ + ΔΔ*E*_*D*128*A*_).

^2^Energy calculated by MM/PBSA.

^3^(calculated - predicted) for energies.

**Table 4:** The decomposition of enthalpy for the eight residues which directly interact with biotin (kcal/mol).

System	S(A)45	D(**A)128**	N23	S27	Y43	N49	S88	T90	biotin
WT	−7.46	−14.00	−5.80	−18.31	−18.46	−15.44	−11.73	−5.30	50.27
S45A	−4.17	−13.90	−5.77	−16.22	−17.34	−19.45	−11.81	−3.95	50.12
D128A	−8.23	−0.20	0.27	−16.86	−17.53	−14.32	−14.58	−4.94	46.11
DM	−5.02	−0.15	0.23	−16.92	−17.71	−12.63	−12.23	−5.46	43.47
Cooperativity	−0.08	−0.05	−0.07	−2.16	−1.31	5.70	2.43	−1.87	−2.20
