# Supplementary material for: The origin of the cooperativity in the streptavidin-biotin system: A computational investigation through molecular dynamics simulations
Source: Sci Rep. 2016 Jun 1;6:27190. doi: 10.1038/srep27190 (PMC4888747; doi:10.1038/srep27190)
Supplement: Supplementary Information [file srep27190-s1.pdf]

Supplementary Information for "The origin of the cooperativity in the streptavidin-biotin system: A computational investigation through molecular dynamics simulations"

Fengjiao Liu[1], **John Z.H. Zhang**[2,3], **and Ye Mei**[1,3,*]

[1]State Key Laboratory of Precision Spectroscopy, School of Physics and Materials Science, East China Normal University, Shanghai 200062, China
[2]Department of Physics, School of Physics and Materials Science, East China Normal University, Shanghai 200062, China
[3]NYU-ECNU Center for Computational Chemistry at NYU Shanghai, Shanghai 200062, China
[*]ymei@phy.ecnu.edu.cn

Table S1. The average distance of hydrogen bonds between eight important residues and biotin in the simulation (Å).

System	S45	D128	N23	S27	Y43	N49	S88	T90
WT	3.1	2.8	3.1	2.6	2.6	2.8	3.2	3.2
S45A		2.8	3.1	2.6	2.7	2.8	3.1	3.1
D128A	3.0		7.4	2.6	2.6	2.8	2.8	2.8
DM			7.1	2.6	2.6	2.9	3.0	3.0

Table S2. The average distance between the carboxyl carbon in biotin and the nitrogen atom in the side chain of N49 during the MD (Å).

System	distance
WT	5.6
S45A	4.9
D128A	5.9
DM	5.7

Table S3. The energy components of pairwise decomposition for the eight residues which directly interact with biotin (kcal/mol).

	System	VDW	ELE	PBSOL	PBTOT
S(A)45	WT	-1.96	-6.18	0.68	-7.46
	S45A	-1.48	-2.31	-0.39	-4.17
	D128A	-1.69	-7.05	0.51	-8.23
	DM	-1.73	-2.82	-0.47	-5.02
	Cooperativity	-0.53	0.36	0.10	-0.08
D(A)128	WT	-0.21	7.73	-21.53	-14.00
	S45A	-0.35	7.99	-21.55	-13.90
	D128A	-0.14	-0.20	0.15	-0.20
	DM	-0.13	-0.13	0.12	-0.15
	Cooperativity	0.15	-0.20	-0.01	-0.05
N23	WT	-0.73	-4.53	-0.54	-5.80
	S45A	-0.72	-4.50	-0.56	-5.77
	D128A	-0.19	1.78	-1.32	0.27
	DM	-0.24	1.61	-1.14	0.23
	Cooperativity	-0.05	-0.21	0.20	-0.07
S27	WT	2.85	-21.38	0.22	-18.31
	S45A	2.24	-18.61	0.17	-16.22
	D128A	2.47	-20.41	1.11	-16.86
	DM	2.54	-20.20	0.75	-16.92
	Cooperativity	0.69	-2.56	-0.32	-2.16
Y43	WT	1.00	-19.18	-0.29	-18.46
	S45A	0.55	-17.67	-0.21	-17.34
	D128A	1.45	-19.13	0.15	-17.53
	DM	0.99	-18.61	-0.11	-17.71
	Cooperativity	-0.01	-0.99	-0.33	-1.31
N49	WT	-0.21	-27.86	12.63	-15.44
	S45A	-0.08	-32.31	12.94	-19.45
	D128A	-0.41	-24.71	10.81	-14.32
	DM	-1.02	-22.69	11.07	-12.63
	Cooperativity	-0.74	6.47	-0.04	5.70
S88	WT	0.73	-19.29	6.83	-11.73
	S45A	0.71	-19.28	6.77	-11.81
	D128A	1.30	-23.39	7.52	-14.58
	DM	0.86	-20.13	7.04	-12.23
	Cooperativity	-0.43	3.26	-0.41	2.43
T90	WT	-0.91	-2.63	-1.76	-5.30
	S45A	-0.64	-1.95	-1.35	-3.95
	D128A	-0.95	-2.18	-1.76	-4.94
	DM	-0.82	-2.84	-1.80	-5.46
	Cooperativity	-0.14	-1.34	-0.45	-1.87
biotin	WT			50.57	50.57
	S45A			50.12	50.12
	D128A			46.11	46.11
	DM			43.47	43.47
	Cooperativity			-2.20	-2.20
